# A Melanoma Brain Metastasis with a Donor-Patient Hybrid Genome following Bone Marrow Transplantation: First Evidence for Fusion in Human Cancer

**DOI:** 10.1371/journal.pone.0066731

**Published:** 2013-06-26

**Authors:** Rossitza Lazova, Greggory S. LaBerge, Eric Duvall, Nicole Spoelstra, Vincent Klump, Mario Sznol, Dennis Cooper, Richard A. Spritz, Joseph T. Chang, John M. Pawelek

**Affiliations:** 1 Deptartment of Dermatology, Yale School of Medicine, New Haven, Connecticut, United States of America; 2 The Yale Cancer Center, Yale School of Medicine, New Haven, Connecticut, United States of America; 3 Human Medical Genetics and Genomics Program, University of Colorado School of Medicine, Aurora, Colorado, United States of America; 4 Denver Police Department Crime Lab, Denver, Colorado, United States of America; 5 Department of Medicine, University of Colorado School of Medicine, Aurora, Colorado, United States of America; 6 Medical Oncology, Yale School of Medicine, New Haven, Connecticut, United States of America; 7 Statistics Department, Yale University, New Haven, Connecticut, United States of America; National Institutes of Health, United States of America

## Abstract

**Background:**

Tumor cell fusion with motile bone marrow-derived cells (BMDCs) has long been posited as a mechanism for cancer metastasis. While there is much support for this from cell culture and animal studies, it has yet to be confirmed in human cancer, as tumor and marrow-derived cells from the same patient cannot be easily distinguished genetically.

**Methods:**

We carried out genotyping of a metastatic melanoma to the brain that arose following allogeneic bone-marrow transplantation (BMT), using forensic short tandem repeat (STR) length-polymorphisms to distinguish donor and patient genomes. Tumor cells were isolated free of leucocytes by laser microdissection, and tumor and pre-transplant blood lymphocyte DNAs were analyzed for donor and patient alleles at 14 autosomal STR loci and the sex chromosomes.

**Results:**

All alleles in the donor and patient pre-BMT lymphocytes were found in tumor cells. The alleles showed disproportionate relative abundances in similar patterns throughout the tumor, indicating the tumor was initiated by a clonal fusion event.

**Conclusions:**

Our results strongly support fusion between a BMDC and a tumor cell playing a role in the origin of this metastasis. Depending on the frequency of such events, the findings could have important implications for understanding the generation of metastases, including the origins of tumor initiating cells and the cancer epigenome.

## Introduction

Tumor cell fusion with motile leucocytes such as myeloid lineage cells or stem cells has been put forward as a unifying explanation for metastasis [1]–[4]. More than a century ago Aichel proposed that cancer might result from fusion between motile leucocytes and other somatic cells, with the qualitative differences between chromosomes causing the hybrid to be “thrown out of the path of the mother cells to form what has come to be known as a malignant cell” [5]. Cancer cell fusion was first detected when human glioblastoma cells were implanted into hamsters and metastases developed with a human-hamster karyotype [6]. This was followed by reports from many laboratories of cancer cell fusion in culture and mice [1]–[3]. More recently, when poorly metastatic Cloudman S91 mouse melanoma cells were fused with normal mouse or human macrophages in culture, hybrids implanted in mice showed high rates of metastasis with decreased survival times of the hosts compared to those of the control melanoma cells used as fusion partners [7]. Metastatic hybrids were highly pigmented, characteristic of the melanocyte lineage, and also expressed numerous myeloid lineage traits such as enhanced chemotactic motility, autophagy and macrophage-like glycosylation patterns [8]–[10]. When human macrophages were used as fusion partners with mouse melanoma cells, hybrids expressed both human and mouse SPARC genes, indicating that the epigenomes of both fusion partners were activated [11]. Similarly, when fluorescent-labeled mouse bone marrow-derived cells were introduced through parabiosis into mice with intestinal tumors, macrophage-cancer cell hybrids formed that expressed transcriptomes characteristic of both parental fusion partners [12]. Likewise, implantation of human glioblastoma or Hodgkins lymphoma cells into hamster cheeks resulted in metastatic human-hamster hybrids with co-expression of human and hamster genes [13]–[14]. Thus, mouse-mouse, human-mouse, and human-hamster hybrids all co-expressed cancer cell and normal cell genes and showed enhanced metastatic capabilities. In other studies, fusion of cancer cells with BMDC's in culture induced aneuploidy, drug resistance, increased invasiveness and tumor heterogeneity [15]–[20]. In human melanomas, “stealth” melanoma cells were detected in lymph node metastases that showed properties consistent with being fusion hybrids [21]. Circulating tumor cells captured from the blood of patients with melanoma, pancreatic and colorectal cancers co-expressed carcinoma and leucocytic markers suggesting BMDC-tumor cell fusion [22].

However, fusion and genomic hybridization have yet to be proven on a genetic basis in human cancer, since genomic differences between cells from the same patient cannot be readily distinguished. To circumvent this problem we have analyzed secondary malignancies arising post allogeneic bone marrow transplants (BMT). In two previous reports we demonstrated donor alleles in patient cancer cells, but there was no provision to identify patient alleles and fusion could thus not be proven [23]–[24]. Here we used STR length-polymorphisms and forensic genetic techniques to analyze genomic DNA in a melanoma brain metastasis from a patient who had previously received a BMT from his brother. The results demonstrate the presence of donor and patient alleles in cancer cells throughout the tumor, indicating that a BMDC-cancer cell fusion event had initiated the generation of this tumor. Pathology analyses and mathematical modeling of allelic patterns supported this conclusion.

## Methods

### Ethics statement

All samples used in this study were preexisting and de-identified before being received by the Yale research team. Exemption was granted under Yale IRB protocol #070900309 (JP and RL) from the Yale University Human Research Protection Program, Institutional Review Board.

### Source of tissues

The patient was a 68-year-old man who received an allogeneic BMT from his brother for treatment of B-cell lymphoma. His last engraftment/chimerism profile was Recipient 3%; Donor 97%. Six years later the patient was diagnosed with metastatic melanoma involving lymph nodes, liver and brain, derived from an unknown primary tumor. We analyzed a brain metastasis (designated “MH3”) consisting of a 0.5×0.2×0.3 cm formalin-fixed paraffin-embedded (FFPE) tissue. The tumor was surgically removed, fixed in formalin, and embedded in paraffin by standard histological procedures. Pre-transplant donor and patient lymphocytes were stored at −90°C in the Yale-New Haven Hospital Stem Cell Bank and retrieved after the tumor analyses were completed.

### Laser Microdissection

Handling and processing of tissue samples was carried out using ultraclean, DNA-free equipment. Five μ-thick histological sections were cut and immunostained for LCA/CD45 (clone 2B11+PD7/26, Dako, catalog N1514) using an autostainer (DAKO, Carpinteria, CA) at the Yale Dermatopathology Laboratories. The antibody was tested for staining efficiency as described in File S1. Tumor cells were microdissected free of LCA/CD45-positive cells from 9 tumor regions using an Arcturus XT laser dissection microscope system. Each sample consisted of dissected tumor cells pooled from one or more areas of the same section.

### DNA extraction

DNA extraction and STR analyses were by the Denver Police Department Crime Laboratory DNA Unit using standard forensic operating and valided procedures [25]. Samples were collected into GeneAmp thin-walled reaction tubes (0.5 ml; Applied Biosystems, Carlsbad, CA, USA). Lymphocyte DNA was extracted on the BioRobot EZ1 platform with the EZ1 DNA Investigator kit trace DNA protocol (Qiagen in USA). Total human and male DNA were assessed with the Quantifiler Duo DNA Quantification Kit (Applied Biosystems, Carlsbad, CA, USA). Two DNA extraction procedures were used. For tumor samples 1–4, DNA was extracted using the 5% Chelex with proteinase K procedure [26]. For samples 5–9, DNA was extracted using the RecoverAll Total Nucleic Acid Isolation Kit for FFPE Tissues (Applied Biosystems, Carlsbad, CA, USA), which improved the DNA yield approximately 5-fold (not shown). The number of tumor cells microdissected per sample was automatically recorded by the Arcturus XT system.

### PCR amplification

PCR was performed with the AmpFlSTR Identifiler PCR Amplification Kit (Applied Biosystems, Carlsbad, CA, USA). Generally, 1 ng of total DNA was targeted in each PCR amplification. In samples with less DNA (<0.1 ng/µl), samples were concentrated 5–20 fold using a Microcon centrifugal filter (Ultracel Ym-100, Millipore, Billerica, MA, USA).

### Forensic genetic analyses of STR loci

Each STR locus was selected to be neutral with respect to other genetic linkage or associations with either Mendelian or non-Mendelian disorders. The loci were polymorphic and exhibited acceptable levels of heterozygosity, typically 70% or higher. They could be assayed together as a PCR multiplex and were robust for degraded DNA [25]. Genotyping of PCR products and interpretation of Short Tandem Repeat-STR alleles were performed using capillary electrophoresis on an ABI Prism 3130 Genetic Analyzer with GeneMapper ID Software version 3.2 (Applied Biosystems, Carlsbad, CA, USA). X and Y chromosomes were detected using the amelogenin assay concurrent with the autosomal STR analyses [25]. Qualitative and quantitative signal-to-noise thresholds were determined with the ABI Identifiler Kit. All peaks >50 relative fluorescence units were scored as true alleles based on a) height and b) peak morphology [25].

### Allelic stutter

Allele signal peaks may overlap technical “stutter” positions from other alleles. Validation studies have established interpretation guidelines for forensic markers to distinguish true allele signals from stutter for any given allele at any locus [27]–[28]. All allele calls deemed significant in this study conformed to these guidelines and were not stutter overlaps.

### Statistical Modeling and Analysis

Bayesian statistical models of fusion and donor cell contamination were fitted and compared using Markov chain Monte Carlo methods [29] implemented using JAGS [30] and R [31] software as described in File S2.

## Results

### Pathology analyses

We performed extensive pathology analyses to ensure that laser dissected tumor samples were not contaminated by infiltrating donor leukocytes. To detect leucocytes we used an antibody to leucocyte common antigen (LCA/CD45), expressed on the surface of mature leukocytes and hemopoietic progenitor cells. In positive control experiments the antibody showed >99% staining efficiency against test cases of dermatitis and lymphoma (Figs. S1 and S2, Table S1 in File S1).

**Figure 1:**
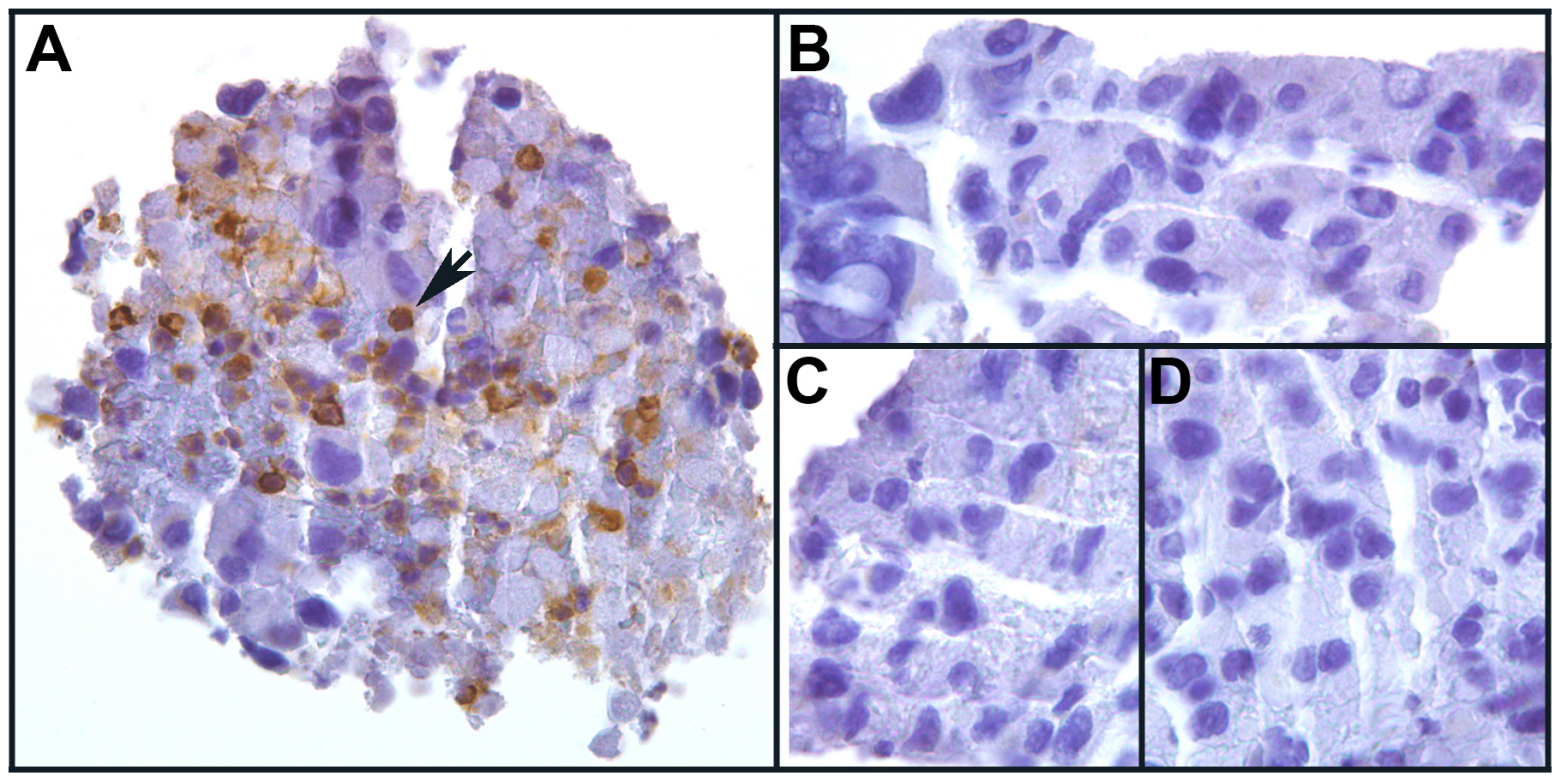
A section of the MH3 melanoma brain metastasis stained for LCA/CD45 (brown chromogen) and counterstained with hematoxylin (blue). A. An area with brown LCA/CD45-positive leucocytes (arrow) intermixed with blue LCA/CD45-negative cancer cells. B-D. Adjacent areas from the same section containing only blue LCA/CD45-negative cancer cells.

**Figure 2:**
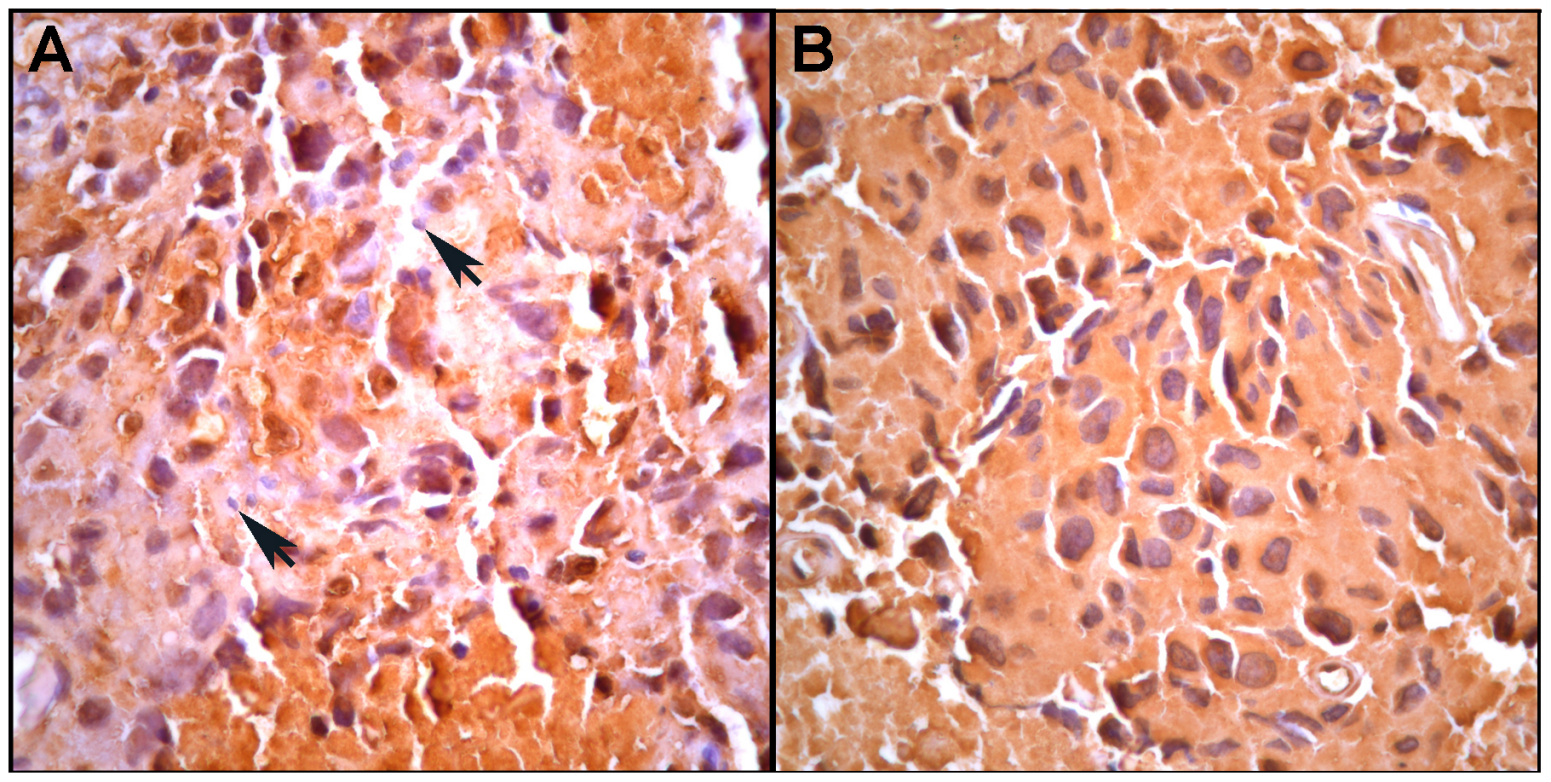
An adjacent section to that in Fig. 1 stained for the melanoma-specific antigen S100. A. An area of S100-positive tumor cells admixed with infiltrating S100-negative leucocytes (arrows). B. An area containing only S100-positive tumor cells. More detailed pathology analyses are presented in Figs S3 and S4 in File S1.

Staining of the MH3 melanoma for LCA/CD45 revealed that some regions contained LCA/CD45-positive leucocytes intermixed with LCA/CD45-negative melanoma cells (Fig. 1A). These were deemed not suitable for laser microdissection. However, other areas contained pure populations of LCA/CD45-negative melanoma cells in groups of tens to hundreds that were suitable for microdissection (Fig. 1B–D). Similarly, when the tumor was stained for S100 to detect melanoma cells [32], some tumor regions were infiltrated with S100-negative leucocytes while others contained only S100-positive melanoma cells (Fig. 2A and B). Thus, two different immunochemistry analyses showed that the MH3 tumor contained areas of virtually pure malignant melanoma cells that could be cleanly microdissected with <1% contaminating leucocytes. A more detailed analysis of S100 staining is presented in Figs. S3 and S4 in File S1.

### Laser microdissection and STR analyses

**Figure 3:**
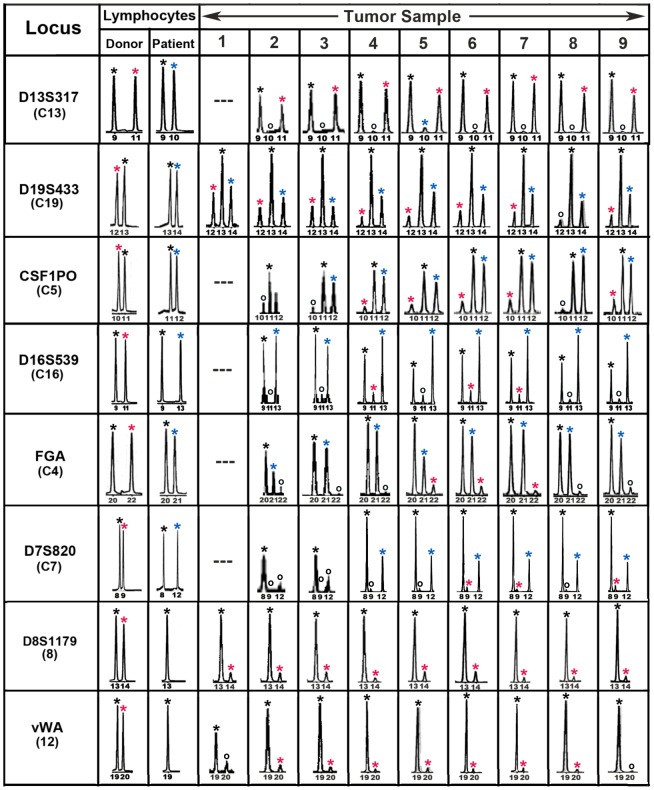
Forensic STR analyses of the MH3 melanoma along with donor and patient pre-BMT lymphocytes. Shown are “informative” loci exhibiting donor and patient specific alleles in pre-BMT lymphocytes. Tumor loci are listed in order of relative abundance of the donor-specific alleles (red asterisk) compared to patient-specific (blue asterisk) and shared alleles (black asterisk). Allele peaks <50 relative fluorescence units were censored as “no call” (open circles). Loci with no detectable alleles after PCR amplification (—).

Tumor sections were stained with LCA/CD45 prior to laser dissection and tumor cells were dissected free of LCA/CD45-positive leucocytes. Tumor cells were isolated from 9 regions throughout the tumor (samples 1–9) and DNA was extracted and amplified for alleles at 14 STR loci. All alleles found in the pre-transplant donor and patient blood lymphocytes were also detected in tumor cells. For each locus there was at least one allele common to both the donor and patient, consistent with the fraternal relationship. Eight loci exhibited donor- specific alleles and six of these exhibited both donor and patient specific-alleles. This indicated that the tumor cells were donor-patient hybrids (Fig. 3, Table 1). Notably, the tumor allelic ratios differed between loci, but for any given locus the allelic ratios were similar in all 9 regions of the tumor. This suggested a relatively stable genotype and likely clonal origin of the metastasis. The remaining loci were uninformative regarding fusion with only shared or patient specific alleles and no donor alleles (Table 2; Fig. S5 in File S1).

**Table 1:** STR loci with donor (D), patient-specific (P) and shared (S) alleles.

Locus	Chromosome	Donor*	Patient*	Tumor*	Tumor Genotype
D13S317	13	9,11	9,10	9,11	D/S
D19S433	19	12,13	13,14	12,13,14	D/P/S
CSF1PO	5	10,11	11,12	10,11,12	D/P/S
D16S539	16	9,11	9,13	9,11,13	D/P/S
FGA	4	20,22	20,21	20,21,22	D/P/S
D7S820	7	8,9	8,12	8,9,12	D/P/S
D8S1179	8	13,14	13,13	13,14	D/S
vWA	12	19,20	19,19	19,20	D/S

* STR units: number of tandem repeats of the locus-specific tetranucleotide sequence. The X and Y chromosomes were detected by the amelogenin assay [25].

**Table 2:** STR loci with only patient-specific (P) and shared alleles.

Locus	Chromosome	Donor*	Patient*	Tumor*	Tumor Genotype
n.a.	X,Y	X,Y	X,Y	X,Y	X,Y
D18S51	14	14,14	14,20	14,20	P/S
TH01	11	7.9.3	7.9.3	7.9.3	S/S
D21S11	2	28,29	28,29	28,29	S/S
D2S1338	2	20,26	20,26	20,26	S/S
TPOX	2	8,8	8,8	8,8	S/S
D5S818	5	12,13	12,13	12,13	S/S

* STR units: number of tandem repeats of the locus-specific tetranucleotide sequence. The X and Y chromosomes were detected by the amelogenin assay [25].

**Figure 4:**
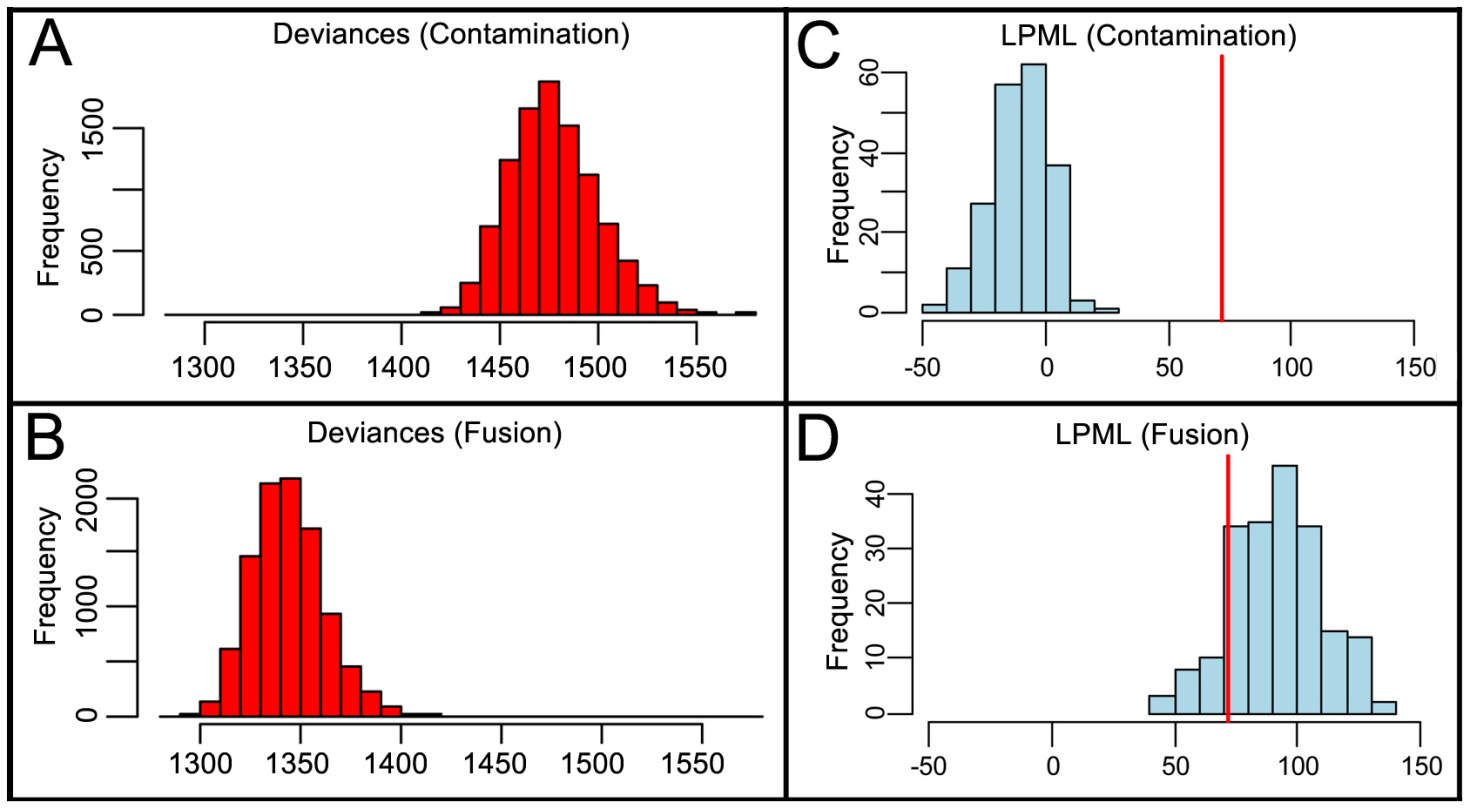
Model comparisons using Markov chain Monte Carlo analyses of allelic data. Panels A and B: Deviances under contamination and fusion models; smaller deviances indicate better fit. Panels C and D: A calibration procedure shows the observed LPML difference (red lines) is rare under the contamination model but typical under the fusion model. More detailed statistical analyses are presented in File S2.

Finally, we fit statistical models to compare the likelihood of donor BMDC-tumor cell fusion versus donor leucocyte contamination (Fig. 4), described in detail in Figs. S6, S7, S8, S9, Table S2 in File S2. The fusion model fit the data better, as shown by its smaller deviances (Fig. 4A and B) and its higher log pseudo-marginal likelihood (LPML), which exceeded the LPML for the contamination model by a difference of 71.4 (Fig. 4C and D, red line). A calibration procedure [33]–[34] to assess the significance of this difference yielded probabilities *P*<0.005 for contamination and *P*>0.3 for fusion (Figs. 4C and D), showing that the observed LPML difference is very rare under the contamination model but not rare under the fusion model. Thus these data strongly support fusion over donor cell contamination as the explanation for the observed allelic dosages.

## Discussion and Conclusion

STR analyses of the tumor DNA revealed that donor and patient alleles were present together at multiple loci and that there were widespread allelic imbalances and aneuploidy. One drawback was the inability to perform STR analyses on individual tumor cells, but for this tumor the lower limit for DNA recovery for STR analyses was about 500 cells, even using the DNA extraction procedure for FFPE cells that improved DNA recovery. Thus, we could not definitively rule out that the patterns might be due to a chimeric mixture of patient tumor cells and donor BMDCs. However this is unlikely for the following reasons: 1) For a given locus the allelic ratios were similar throughout the tumor. It is difficult to explain these repeating patterns as due to chimeric mixtures as this would require that the different cell types existed together in the same ratios throughout the tumor. Notably, while most of the informative tumor loci had patient and shared alleles in greater relative abundance to donor-specific alleles, tumor locus D13S317 was reversed, with the patient-specific allele absent and the donor-specific allele in prominence. Since the initial dose of genomic DNA for each sample was determinative of PCR product intensity and varied widely between samples, given the consistency in allelic ratios from sample to sample the reversal of DNA dosage at locus D13S317 cannot be explained by preferential PCR or leukocyte contamination. 2) The tumor cells were dissected from regions free of LCA-positive cells (Fig. 1). 3) It is unlikely that the results were due to contaminating DNA from exogenous sources. Any contaminating DNA would have had to come from the donor or patient since all the alleles detected in the tumor cells could be accounted for in pre-transplant lymphocytes from one or the other of these individuals. One source of such contamination could be the lymphocytes themselves as they contained vast amounts of DNA compared to those in the tumor samples. However, this was ruled out because the lymphocytes were retrieved from long-term storage in liquid N_2_ only after the tumor analyses were completed. Also, had there been a pervasive source of contaminating DNA in the tumor, it should have been present at similar levels in the necrotic areas and this was not the case, as discussed above. 4) Our statistical analyses of the allelic patterns strongly favored BMDC-melanoma cell fusion over leucocyte contamination models.

In stem cell biology both transdifferentiation and fusion appear to be operative in the transformation of stem cells into differentiated somatic cells [35]–[38]. However the origin of cancer stem cells/tumor initiating cells is controversial. In two previous reports of secondary malignances arising in patients post allogeneic bone marrow transplant, donor genes were found in tumor cells, strongly suggesting fusion [23]–[24]. But in neither case were patient-specific genes also identified in the tumor cells and alternative mechanisms to fusion could not be ruled out, such as transdifferentiation of donor stem cells into cancer cells. However in the tumor described herein, forensic STR analyses revealed that both donor and patient alleles were present in the tumor cells throughout and the tumor appeared to consist largely if not solely of BMDC-tumor cell hybrids. Moreover, the repeating allelic patterns for each locus throughout the tumor indicated a clonal origin of the metastasis and suggested that the tumor was generated from a prior fusion event between a donor BMDC and a patient tumor cell. Thus, at least in this case, we conclude that the tumor-initiating cell was a BMDC-tumor cell hybrid. The extent to which this mechanism is operative in other tumors remains to be determined.

In earlier studies, experimental tumor hybrids generated *in vitro* between cancer cells, including melanoma, and normal epithelial cells or fibroblasts were generally suppressed in tumorigenicity and the expression of differentiated functions, leading to the discovery of tumor suppressor genes [39]–[40]. But later, using macrophages as fusion partners with melanoma cells, resultant hybrids expressed genes and differentiated traits from both parents and metastasis was markedly enhanced [7]–[11]. This indicated co-expression of epigenomes from both parental lineages. Co-expressed hybrid genomes could account for the complexity of gene expression patterns in cancer cells and also how malignant cells could have such a large repertoire of myeloid-like capabilities such as angiogenesis, matrix alterations, motility, chemotaxis and immune signaling pathways, as well as undergo epidermal-mesodermal transition [1], [41].

**Figure 5:**
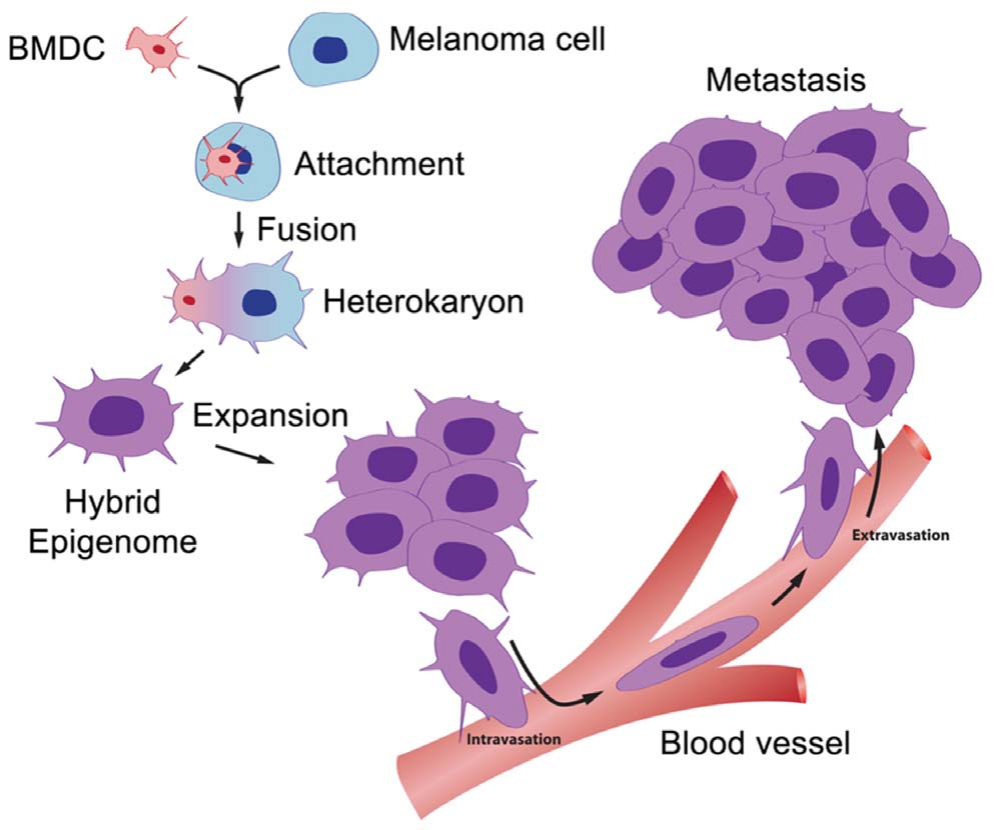
The BMDC-cancer cell fusion hypothesis. A motile BMDC (red) such as a macrophage or stem cell is drawn to a cancer cell (blue). The outer cell membranes of the two cells become attached. Fusion occurs with the formation of a bi-nucleated heterokaryon having a nucleus from each of the fusion partners. The heterokaryon goes through genomic hybridization creating a melanoma-BMDC hybrid with co-expressed epigenomes, conferring deregulated cell division and metastatic competence to the hybrid.

A model for metastasis resulting from fusion of bone marrow-derived cells is diagramed schematically in Figure 5. While this has largely been verified in animal tumor models and cell culture, evidence for fusion in human cancer has heretofore been lacking. Our findings show for the first time in a human cancer that generation of a metastasis and acquisition of its aberrant genetic patterns resulted from fusion and genomic hybridization between a BMDC and a cancer cell. Depending on the frequency of such events, the findings could have important implications for understanding metastasis, including the origins of tumor initiating cells and the cancer epigenome.

## Supporting Information

File S1 Figures S1, S2, S3, S4, S5 and Table S1.
(PDF)

File S2 Figures S6, S7, S8, S9 and Table S2.
(PDF)

## References

[1] Pawelek JM (2000) Tumor Cell Hybridization and Metastasis Revisited. Melanoma Res 10: 507–514. 11198471 10.1097/00008390-200012000-00001

[2] Pawelek JM, Chakraborty AK (2008) Fusion of tumour cells with bone marrow-derived cells: a unifying explanation for metastasis. Nat Rev Cancer 8: 377–386. 18385683 10.1038/nrc2371

[3] Pawelek JM, Chakraborty AK (2008) The cancer cell-leukocyte fusion theory of metastasis. Adv Cancer Res 101: 397–444. 19055949 10.1016/S0065-230X(08)00410-7

[4] Duelli D, Lazebnik Y (2007) Cell-to-cell fusion as a link between viruses and cancer. Nat Rev Cancer 7: 968–976. 18034186 10.1038/nrc2272

[5] Aichel O (1911) *Vorträge und Aufsätze über Entvickelungsmechanik Der Organismen*, “Über Zellverschmelzung mit Qualitativ Abnormer Chromosomenverteilung als Ursache der Geschwulstbildung” *in* Roux W, ed. [About cell fusion with qualitatively abnormal chromosome distribution as cause for tumor formation.] Leipzig, Germany: Wilhelm Engelmann, Chapter XIII pp. 92–111.

[6] Goldenberg DM, Pavia RA, Tsao MC (1974) *In vivo* hybridization of human tumour and normal hamster cells. Nature 250: 649–651. 4859359 10.1038/250649a0

[7] Rachkovsky MS, Sodi S, Chakraborty A, Avissar Y, Bolognia J, et al (1998) Enhanced metastatic potential of melanoma×macrophage fusion hybrids. Clin. Exp. Metastasis 16: 299–312. 10.1023/a:1006557228604 9626809

[8] Rachkovsky M, Pawelek J (1999) Acquired melanocyte stimulating hormone-inducible chemotaxis following macrophage fusion with Cloudman S91 melanoma cells. Cell Growth Differ 10: 515–524. 10437919

[9] Chakraborty AK, Sodi S, Rachkovsky M, Kolesnikova N, Platt JT, et al (2000) A spontaneous murine melanoma lung metastasis comprised of host×tumor hybrids. Cancer Res 60: 2512–2519. 10811133

[10] Pawelek JM, Chakraborty AK, Rachkovsky ML, Orlow SJ, Bolognia JL, et al (2000) Altered N-glycosylation in macrophage×melanoma fusion hybrids. Cell. Mol. Biol 45: 1011–1027. 10644005

[11] Chakraborty AK, de Freitas Sousa J, Espreafico EM, Pawelek JM (2001) Human monocyte×mouse melanoma fusion hybrids express human gene. Gene 275: 103–106. 11574157 10.1016/s0378-1119(01)00647-3

[12] Powell AE, Anderson EC, Davies PS (2011) Fusion between intestinal epithelial cells and macrophages in a cancer context results in nuclear reprogramming. Cancer Res 71: 1497–2005. 21303980 10.1158/0008-5472.CAN-10-3223 PMC3079548

[13] Goldenberg DM, Zagzag D, Heselmeyer-Haddad KM, Berroa Garcia LY, Ried T, et al (2012) Horizontal transmission and retention of malignancy, as well as functional human genes, after spontaneous fusion of human glioblastoma and hamster host cells in vivo. Int J Cancer 131: 49–58. 21796629 10.1002/ijc.26327 PMC3307948

[14] Goldenberg DM, Gold DV, Loo M, Liu D, Chang CH, et al (2013) Horizontal transmission of malignancy: in-vivo fusion of human lymphomas with hamster stroma produces tumors retaining human genes and lymphoid pathology. PLoS One 8: e55324 Epub 2013 Feb 6. doi:10.1371/journal.pone.0055324 23405135 10.1371/journal.pone.0055324 PMC3566191

[15] Berndt B, Zanker KS, Dittmar T (2013) Cell Fusion is a Potent Inducer of Aneuploidy and Drug Resistance in Tumor Cell/Normal Cell Hybrids. Crit Rev Oncog 18 97–113. 23237554 10.1615/critrevoncog.v18.i1-2.60

[16] Nagler C, Hardt C, Zänker KS, Dittmar T (2011) Co-cultivation of murine BMDCs with 67NR mouse mammary carcinoma cells give rise to highly drug resistant cells. Cancer Cell Int 11: 21. 21711510 10.1186/1475-2867-11-21 PMC3135493

[17] Mercapide J, Anzanello F, Rappa G, Lorico A (2012) Relationship between tumor cell invasiveness and polyploidization. PLoS One 7: e53364 doi:10.1371/journal.pone.0053364 23300919 10.1371/journal.pone.0053364 PMC3534062

[18] Rappa G, Mercapide J, Lorico A (2012) Spontaneous formation of tumorigenic hybrids between breast cancer and multipotent stromal cells is a source of tumor heterogeneity. Am J Pathol 180: 2504–2515. 22542847 10.1016/j.ajpath.2012.02.020 PMC3378856

[19] Mercapide J, Rappa G, Lorico A (2012) The intrinsic fusogenicity of glioma cells as a factor of transformation and progression in the tumor microenvironment. Int J Cancer 131: 334–343. 21858806 10.1002/ijc.26361

[20] Ding J, Jin W, Chen C, Shao Z, Wu J (2012) Tumor associated macrophage×cancer cell hybrids may acquire cancer stem cell properties in breast cancer. PLoS One 7: e41942 doi:10.1371/journal.pone.0041942 22848668 10.1371/journal.pone.0041942 PMC3405038

[21] Itakura E, Huang RR, Wen DR, Cochran AJ (2011) “Stealth” melanoma cells in histology-negative sentinel lymph nodes. Am J Surg Pathol 35: 1657–1665. 21997686 10.1097/PAS.0b013e3182322cf7 PMC3203732

[22] Clawson GA, Kimchi E, Patrick SD, Xin P, Harouaka R, et al (2012) Circulating tumor cells in melanoma patients. PLOS One 7: e41052 doi:10.1371/journal.pone.0041052 22829910 10.1371/journal.pone.0041052 PMC3400630

[23] Chakraborty A, Lazova R, Davies S, Bäckvall H, Ponten F, et al (2004) Donor DNA in a renal cell carcinoma metastasis from a bone marrow transplant recipient. Bone Marrow Transplant 34: 183–186. 15195072 10.1038/sj.bmt.1704547

[24] Yilmaz Y, Lazova R, Qumsiyeh M, Cooper D, Pawelek J (2005) Donor Y chromosome in renal carcinoma cells of a female BMT recipient. Bone Marrow Transplant 35: 1021–1024. 15778726 10.1038/sj.bmt.1704939

[25] Collins PJ, Hennessy LK, Leibelt CS, Roby RK, Reeder DJ, et al (2004) Developmental validation of a single-tube amplification of the 13 CODIS STR loci, D2S1338, D19S433, and amelogenin: the AmpFlSTR Identifiler PCR Amplification Kit. J Forensic Sci 49: 1265–1277. 15568700

[26] Walsh SP, Metzger DA, Huguchi R (1991) Chelex 100 as a Medium for Simple Extraction of DNA for PCR-Based Typing from Forensic Material. Biotechniques 10: 506–513. 1867860

[27] Leclair B, Frégeau CJ, Bowen KL, Fourney RM (2004) Systematic analysis of stutter percentages and allele peak height and peak area ratios at heterozygous STR loci for forensic casework and database samples. J Forensic Sci 49: 968–980. 15461097

[28] Boon LK, Jeevan NH, Primulapathi JK, Othman MI, Hin LY (2006) Int Congress Series 1288, 379–381.

[29] Gamerman D, Lopes HF (2006) Markov chain Monte Carlo: stochastic simulation for Bayesian inference. Vol.68 (Chapman & Hall/CRC).

[30] Plummer M (2003) JAGS: A program for analysis of Bayesian graphical models using Gibbs sampling, in Proceedings of the 3rd International Workshop on Distributed Statistical Computing (Vienna, Austria).

[31] R: A Language and Environment for Statistical Computing (2010) R Foundation for Statistical Computing, Vienna, Austria.

[32] Ohsie SJ, Sarantopoulos GP, Cochran AJ, Binder SW (2008) Immunohistochemical characteristics of melanoma. J Cut Pathol 35: 433–444. 10.1111/j.1600-0560.2007.00891.x 18399807

[33] Vlachos PK, Gelfand AE (2003) On the calibration of Bayesian model choice criteria. J Statistical Planning and Inference 111: 223–234.

[34] Draper D, Krnjajić M (2011) Bayesian Model Specification: Some problems related to model choice and calibration, in Applied Methods of Statistical Analysis, Simulations and Statistical Inference (Novosibirsk, Russia), 133–142.

[35] Zhang S, Wang D, Estrov Z, Raj S, Willerson JT, et al (2004) Both cell fusion and transdifferentiation account for the transformation of human peripheral blood CD34-positive cells into cardiomyocytes in vivo. Circulation 110: 3803–3807. 15596566 10.1161/01.CIR.0000150796.18473.8E

[36] Bjerkvig R, Tysnes BB, Aboody KS, Najbauer J, Terzis AJ (2005) Opinion: the origin of the cancer stem cell: current controversies and new insights. Nat Rev Can 5: 899–904 Erratum in: Nat Rev Cancer 5: 995. 10.1038/nrc1740 16327766

[37] Dittmar T, Seidel J, Zaenker KS, Niggemann B (2006) Carcinogenesis driven by bone marrow-derived stem cells. Contrib Microbiol 13: 156–169. 16627964 10.1159/000092971

[38] Lluis F, Cosma MP (2010) Cell-fusion-mediated somatic-cell reprogramming: a mechanism for tissue regeneration. Cell Physiol 223: 6–13. 10.1002/jcp.22003 20049847

[39] Davidson RL (1971) Regulation of differentiation in cell hybrids. Fed Proc 30: 926–929. 4324980

[40] Harris H (1988) The analysis of malignancy by cell fusion: the position in 1988. Cancer Res 48: 3302–3306. 3370633

[41] Pawelek J (2005) Tumor cell fusion as a source of myeloid traits in cancer. The Lancet Oncol 6: 988–993. 16321767 10.1016/S1470-2045(05)70466-6

