# Supplementary material for: A Melanoma Brain Metastasis with a Donor-Patient Hybrid Genome following Bone Marrow Transplantation: First Evidence for Fusion in Human Cancer
Source: PLoS One. 2013 Jun 26;8(6):e66731. doi: 10.1371/journal.pone.0066731 (PMC3694119; doi:10.1371/journal.pone.0066731)
Supplement: File S1 — Figures S1, S2, S3, S4, S5 and Table S1. (PDF) [file pone.0066731.s001.pdf]

**Lazova et al. A melanoma metastasis with a donor-patient hybrid genome: Forensic evidence for fusion in human cancer**

**Supporting Information File S2**
**Figures S6-S9**
**Table S2**

**Pathology Studies; Additional STR Analyses-Uninformative Loci**

I. Positive controls for the LCA/CD45 antibody.

A. Figure S1: Dermatitis

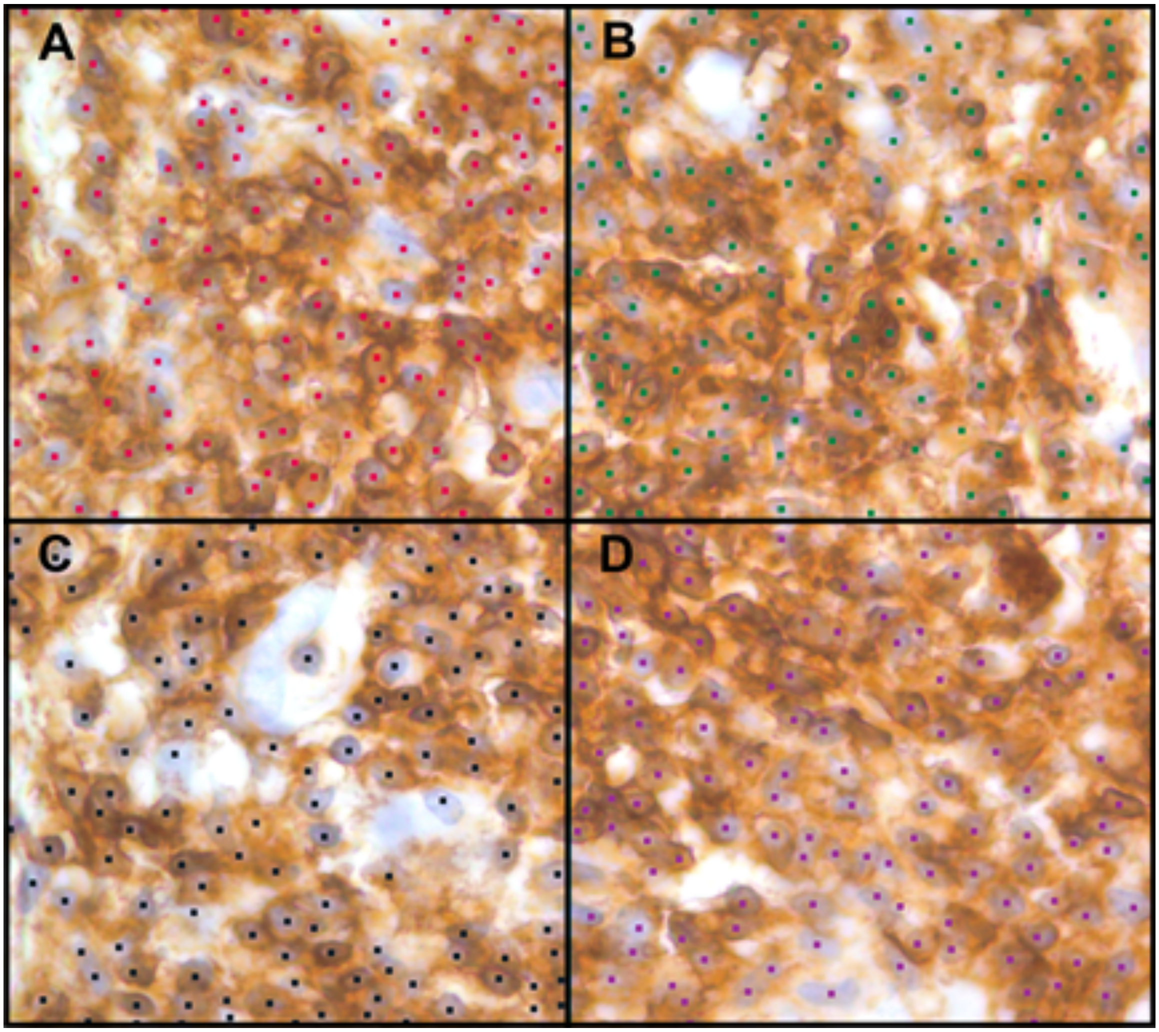


**Figure S1.** Positive control for the LCA/CD45 antibody staining efficency in a test case of dermatitis. Shown is a selected area with high densities of lymphocytes. For ease of scoring, the area was divided into quadrants and once scored the LCA-positive cells were labeled with colored dots. Summarized in Table S1.

I. Positive controls for the LCA/CD45 antibody (Cont'd)

B. Figure S2: Lymphoma

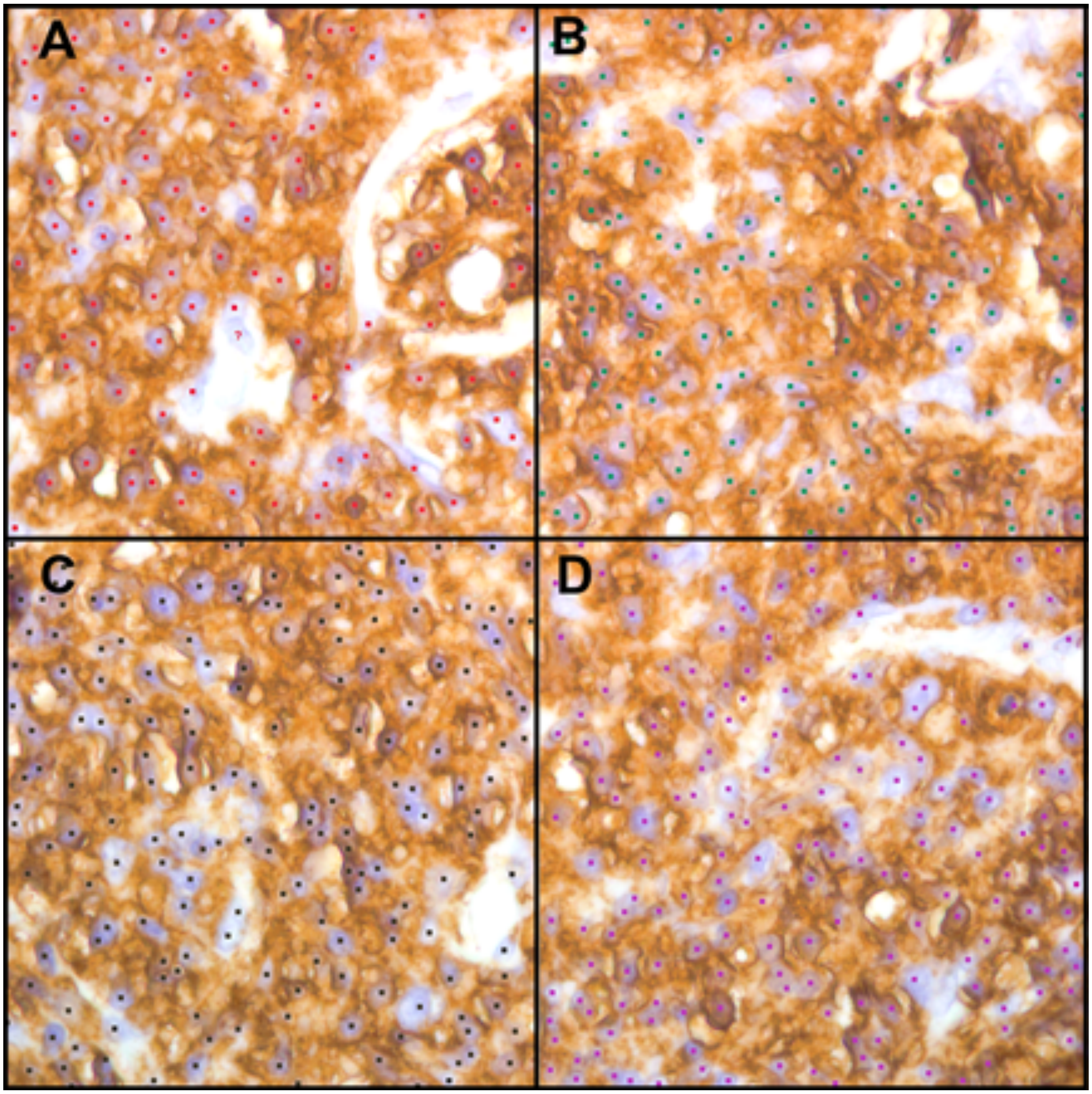


**Figure S2**. Positive control for the LCA/CD45 antibody staining efficiency in a test case of lymphoma. Shown is a selected area with high densities of lymphocytes. LCA-positive cells were labeled with colored dots and scored as described in Figure S1. Summarized in Table S1.

I. Table S1: Postive controls for the LCA/CD45 antibody: LCA-positive cells per circumscribed microscope field

| **Dermatitis** | **LCA$^{pos}$** | **LCA$^{neg}$** | **%LCA$^{pos}$** | **Lymphoma** | **LCA$^{pos}$** | **LCA$^{neg}$** | **%LCA$^{pos}$** |
|---|---|---|---|---|---|---|---|
| **Field** | **Cells/ Field** | | | **Field** | **Cells/ Field** | | |
| A | 133 | 0 | 100 | A | 109 | 1 | 99.1 |
| B | 128 | 0 | 100 | B | 135 | 0 | 100 |
| C | 125 | 0 | 100 | C | 204 | 0 | 100 |
| D | 118 | 0 | 100 | D | 175 | 0 | 100 |
| **Total** | 504 | 0 | 100 | **Total** | 623 | 1 | 99.8 |

*Summary of the positive controls for the LCA/CD45 antibody: staining efficiency in test cases of dermatitis and lymphoma. Slides were stained for LCA/CD45 with mouse anti-human LCA antibody clone 2B11 + PD7/26 (Dako, catalog N1514). Data are from Supporting Figures S1 (dermatitis) and S2 (lymphoma). Slides were stained with brown chromogen and counterstained with hematoxylin (blue). Using Photoshop tools of digital images, fields of high leucocyte density were circumscribed. All cells were scored as to LCA positivity.

II. Supporting pathology studies on leucocyte infiltration in the MH3 melanoma.

Conlusion: Comparing hematoxylin and eosin (H&E) staining followed by re-staining with S100 showed that while some tumor regions were infiltrated with S100-negative leucocyes (Fig. S3), others contained only S100-positive melanoma cells (Fig. S4).

Figure S3
A region of MH3 tumor infiltrated with S100-negative leucocytes

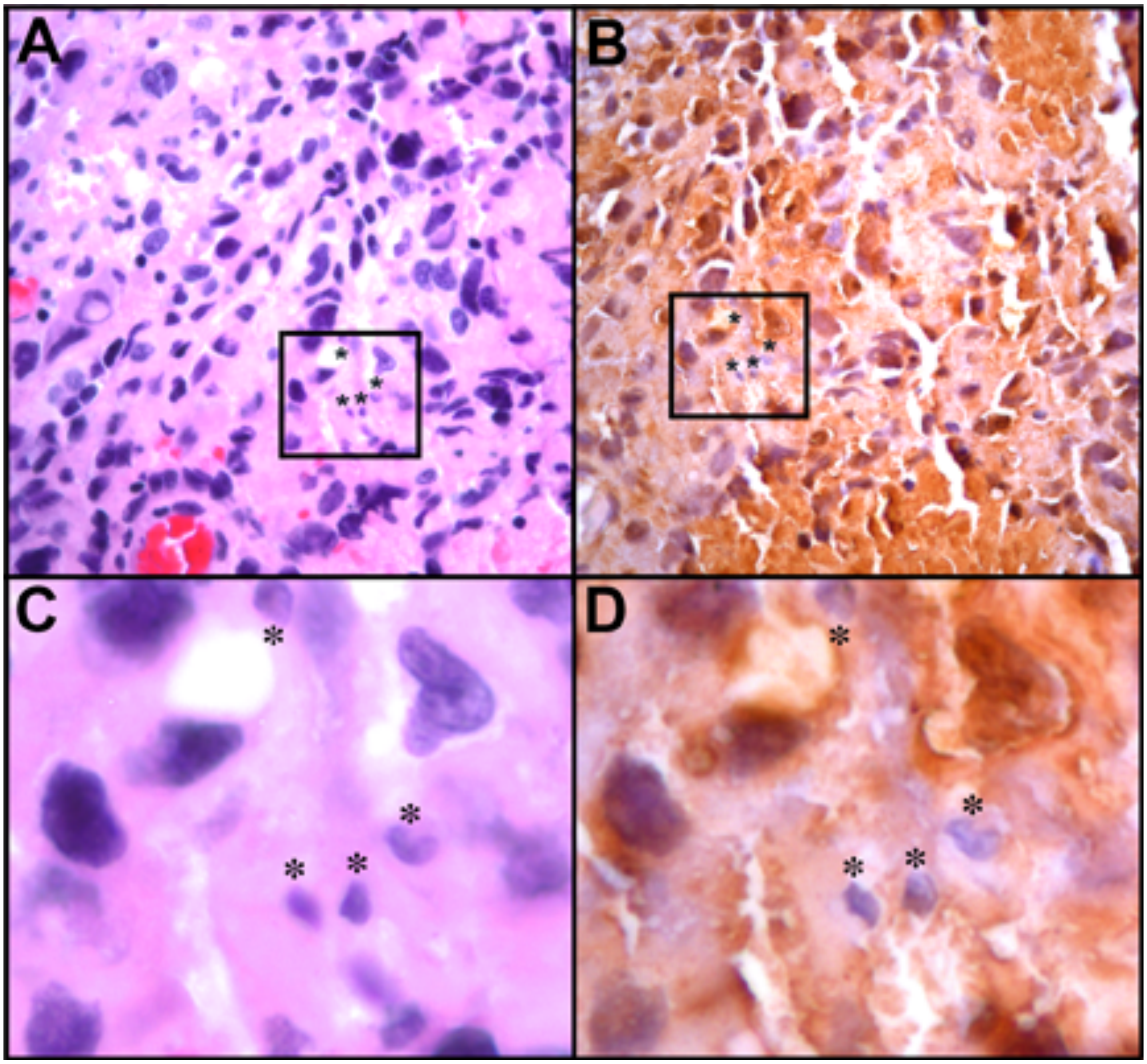


**Figure S3.** A) An H&E stained section containing large and pleomorphic tumor cells with hyperchromatic irregular nuclei along with smaller leucocytes (insert). B) (Insert from A). Four leukocytes, most likely lymphocytes, labeled by asterisks, adjacent to much larger tumor cells with large, dark, and irregular nuclei. C) The same area as (A) after destaining and restaining with the S100 antibody. D) (Insert from C) The lymphocytes are negative for S-100 (D). A and C (20x), B and D (63X)

II. Supporting pathology studies on leucocyte infiltration in the MH3 melanoma (cont'd).

Figure S4
An S100-positive region of the MH3 melanoma free of infiltrating leucocytes.

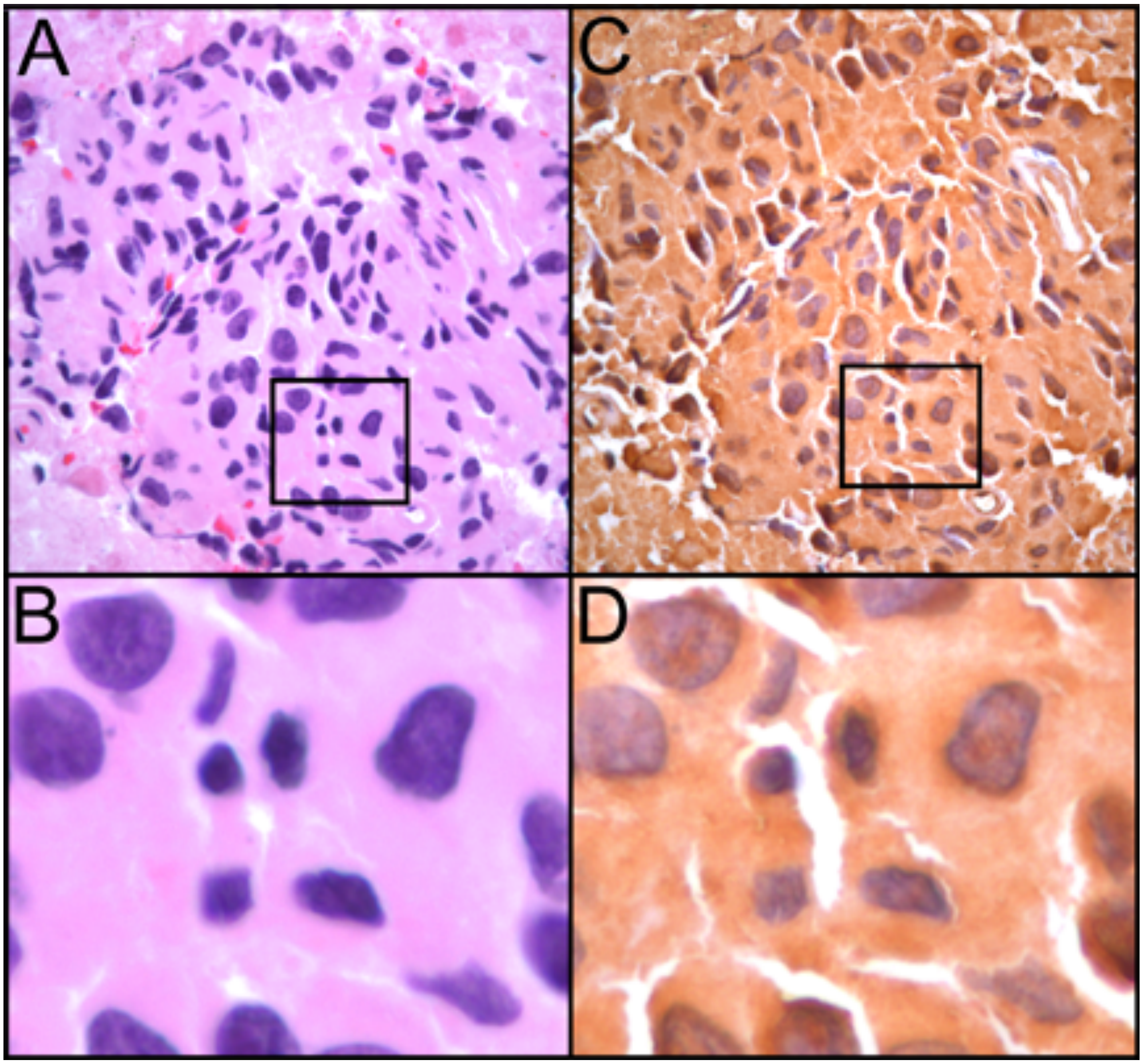


**Figure S4**. An H&E/S100-stained area from the same section as in Fig. S3 containing a pure population of tumor cells with variation in their size and nuclear staining. All tumor cells are positive for S-100. A and C (20x), B and D (63X)

III. Additional forensic genetics studies of MH3 melanoma: Allelic profiles of “non-informative” loci.

Figure S5:

The non-informative loci contained no donor alleles but only shared alleles or in one case a patient specific allele (locus D18S51)

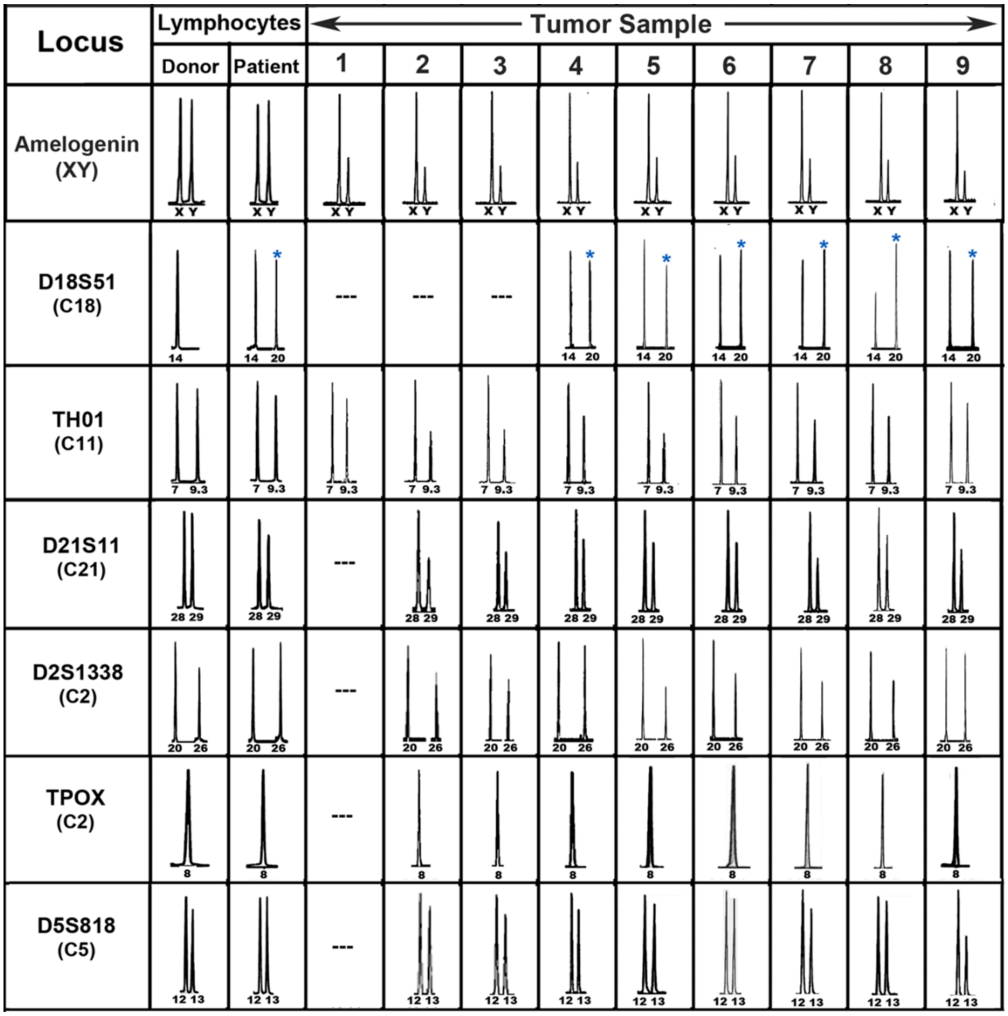


**Figure S5**. Melanoma tumor MH3, “non-informative” loci, no donor-specific STR alleles in pre-transplant blood lymphocytes. The number of repeating STR units is shown beneath each peak. “C” followed by a number designates the chromosome number of the locus. Loci with no detectable alleles after PCR amplification (---).
