# Supplementary material for: A Melanoma Brain Metastasis with a Donor-Patient Hybrid Genome following Bone Marrow Transplantation: First Evidence for Fusion in Human Cancer
Source: PLoS One. 2013 Jun 26;8(6):e66731. doi: 10.1371/journal.pone.0066731 (PMC3694119; doi:10.1371/journal.pone.0066731)
Supplement: File S2 — Figures S6, S7, S8, S9 and Table S2. (PDF) [file pone.0066731.s002.pdf]

Lazova et al. A melanoma metastasis with a donor-patient hybrid genome: Forensic evidence for fusion in human cancer

Supporting Information File 2
Figures S6-S9
Table S2

Statistical Modeling and Analysis

Data

We focused on the six loci having both patient-specific and donor-specific alleles, since they seemed the most informative for the questions considered below. Each of these six loci also happened to have one shared allele. A graphical display of the RFU data for these loci is shown in Fig. S6. Allele peaks that did not reach a height of at least 50 Relative Florescence units (RFU) were not called as 'alleles' and were not scored with numeric values, due to questions about validity below 50 RFU, which in this genotyping system is considered a qualitative threshold for signal to noise reasons. Therefore, our models treated such allelic peaks as left-censored (*1*); that is, only the information that the peak value was less than 50 RFU was used.

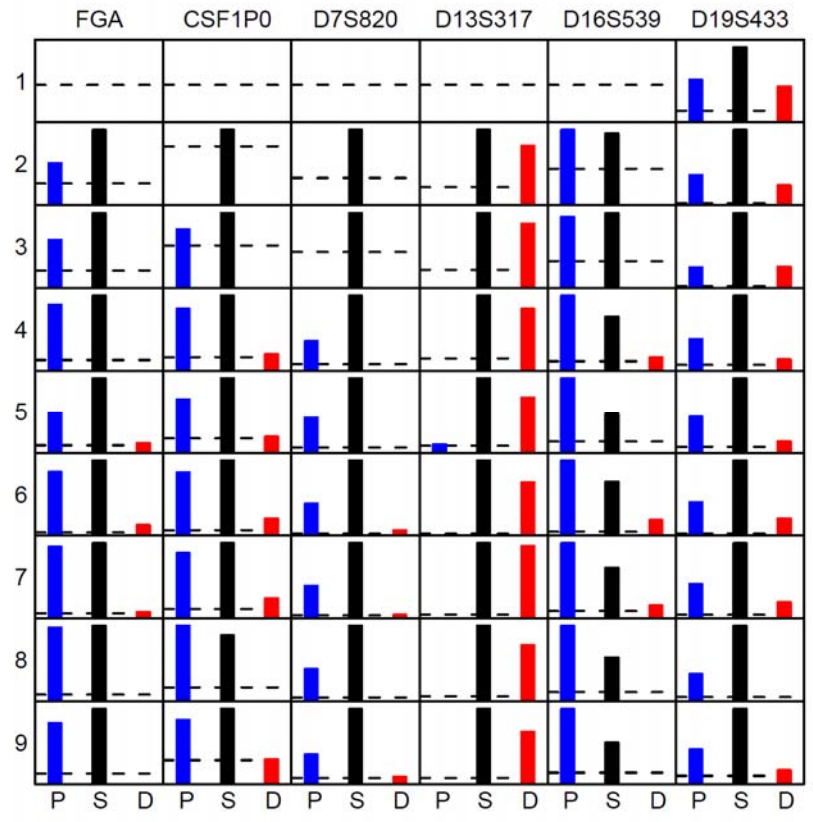

Fig. S6. RFU data for the six tumor loci exhibiting donor specific (red), patient specific (blue) and shared alleles (black). Horizontal dashed lines represent the height of 50 RFU in each plot.

Implausibility of contamination: a first approach

An alternative potential explanation for the presence of donor-specific alleles is that, despite extensive procedural precautions and pathological analyses described in the text, our tumor samples were contaminated by leukocytes, ultimately derived from the donor. However, simple numerical observations suggest this is unlikely. For $s = 1,\ldots,9$, let δ_s denote the *donor contamination* ratio for section s, defined to be the ratio of the number of donor leukocytes to the number of patient cancer cells in section s. A simple indication of contamination levels necessary to explain the donor alleles is given by ratios of donor-specific alleles to patient-specific alleles. Let P_{sl} and D_{sl} denote, respectively, the patient- and donor-specific allelic peaks (measured in RFU) in section s at locus l, and let i_l denote the number of patient-specific alleles at locus l in each patient cancer cell (which may be greater than 1, e.g., due to aneuploidy). Letting n_s denote the number of patient cancer cells in section s, if each of these cells has i_l patient-specific alleles at locus l, there will be a total of $n_s i_l$ patient-specific alleles. By definition, there are also $\delta_s n_s$ donor leukocytes in section s, having 1 donor-specific allele each, and contributing a total of $\delta_s n_s$ donor-specific alleles. So the ratio of counts of donor-specific to patient-specific alleles in section s at locus l is $\frac{\delta_s n_s}{n_s i_l} = \frac{\delta_s}{i_l}$. Therefore, assuming that P_{sl} and D_{sl} are expected to be proportional to the total number of patient- and donor-specific alleles at locus l in section s leads to an expectation that $\frac{D_{sl}}{P_{sl}} \approx \frac{\delta_s}{i_l}$. Thus, in a contamination scenario, if patient-specific alleles are detected (so that $i_l \geq 1$), δ_s is expected to be at least $\frac{D_{sl}}{P_{sl}}$. For example, in section 3 of locus D19S433, the donor-specific and patient-specific RFU's are 575 and 553, respectively, suggesting that $\delta_3 \geq \frac{575}{553} = 104\%$. However, our pathology studies concluded that donor contamination levels like δ_3 are likely less than 1% and very unlikely to reach as high as 5%. Thus, such a high value for δ_3 would not be credible, casting doubt upon a contamination explanation. In a similar way, many other combinations of section and locus argue against contamination.

Statistical Models of fusion and contamination

The preceding considerations suggest that the credibility of donor contamination as an explanation for the donor alleles is low. To provide a more quantitative probabilistic analysis, we assumed that deviations from exact equality in expected approximations such as $\frac{D_{sl}}{P_{sl}} \approx \frac{\delta_s}{i_l}$ may be described by statistical distributions. We fit a number of Bayesian statistical models (*2*) to the data for the six loci showing both patient and donor specific alleles. Here we will report in detail on two models, referred to as "fusion" and

"contamination," which assumed that the donor alleles are explained by fusion and by donor contamination, respectively.

We modeled the RFU intensity for each allele at a given locus and section as a random variable with a Normal distribution whose mean is proportional to the number of such alleles present in the section, and whose variance incorporates both additive and multiplicative random perturbations. To describe the models, let P , S , and D generically denote patient-specific, shared, and donor-specific alleles in genotypes. In the fusion model, the cancer cells are hybrids resulting from fusion, and at locus l we assumed the cancer cells have genotypes of the form $i_l \cdot P + j_l \cdot S + k_l \cdot D$, that is, i_l copies of the patient-specific allele, j_l copies of the shared allele, and k_l copies of the donor-specific allele, where i_l, j_l, and k_l are nonnegative integers. In the contamination model, the patient cancer cells have genotypes of the form $i_l \cdot P + j_l \cdot S$, and the donor alleles in section s are explained by donor contamination at level δ_s arising from blood cells having genotype $1 \cdot S + 1 \cdot D$, where the allelic copy numbers are assumed to be 1 since these are not cancer cells. The fusion model assumes that donor contamination is negligible and the source of the donor alleles is the hybrid cancer cells. Both models allow contamination of the samples by patient non-cancer cells at level π_s, defined as the ratio of the number of patient non-cancer cells (having genotype $1 \cdot P + 1 \cdot S$) to the number of patient cancer cells in section s.

For the fusion model, allelic peak heights P_{sl} , S_{sl} , and D_{sl} are modeled as random variables having Normal distributions with the following means and variances:

$$E(P_{sl}) = \lambda_{sl} \frac{i_l + \pi_s}{i_l + j_l + k_l + 2\pi_s}, \quad \mathrm{var}(P_{sl}) = \sigma^2_{\mathrm{add}} + \left(E(P_{sl})\right)^2 \sigma^2_{\mathrm{mult}},$$
$$E(S_{sl}) = \lambda_{sl} \frac{j_l + \pi_s}{i_l + j_l + k_l + 2\pi_s}, \quad \mathrm{var}(S_{sl}) = \sigma^2_{\mathrm{add}} + \left(E(S_{sl})\right)^2 \sigma^2_{\mathrm{mult}},$$
$$E(D_{sl}) = \lambda_{sl} \frac{k_l}{i_l + j_l + k_l + 2\pi_s}, \quad \mathrm{var}(D_{sl}) = \sigma^2_{\mathrm{add}} + \left(E(D_{sl})\right)^2 \sigma^2_{\mathrm{mult}}.$$

Letting λ_{sl} denote the expected sum of the allelic peaks at locus l in section s , the expressions for the means follow from the assumption that tumor section s consists of a mixture, in proportions 1 to π_s , of cancer cells having genotype $i_l \cdot P + j_l \cdot S + k_l \cdot D$ at locus l and patient noncancer cells having genotype $1 \cdot P + 1 \cdot S + 0 \cdot D$. Together with the assumption that the expected value of an allelic peak should be proportional to the number of copies of that allele in the section, this leads to

$$\left(E(P_{sl}), E(S_{sl}), E(D_{sl})\right) \propto 1 \cdot (i_l, j_l, k_l) + \pi_s \cdot (1,1,0) = \left(i_l + \pi_s, j_l + \pi_s, k_l\right).$$

The expressions for the variances allow the allelic peaks to be subject to additive and multiplicative random perturbations. These expressions arise by assuming, for example, that $P_{sl} = E(P_{sl})\left(1 + \sigma_{\mathrm{mult}} Z^{\mathrm{mult}}_{sl}\right) + \sigma_{\mathrm{add}} Z^{\mathrm{add}}_{sl}$, where Z^{mult}_{sl} and Z^{add}_{sl} are independent

random variables having Normal distributions with mean 0 and variance 1. We refer to σ^2_{add} and σ^2_{mult} as the additive variance and multiplicative variance, respectively.

The contamination model assumes a mixture of patient cancer cells having genotype $i_l \cdot P + j_l \cdot S + 0 \cdot D$, patient noncancer cells having genotype $1 \cdot P + 1 \cdot S + 0 \cdot D$, and donor noncancer cells having genotype $0 \cdot P + 1 \cdot S + 1 \cdot D$, in numbers proportional to 1, π_s, and δ_s in section s. Thus,

$$\big(E(P_{sl}), E(S_{sl}), E(D_{sl})\big) \propto 1 \cdot (i_l, j_l, 0) + \pi_s \cdot (1,1,0) + \delta_s \cdot (0,1,1),$$

and so the above expressions for the means are replaced by

$$E(P_{sl}) = \lambda_{sl} \frac{i_l + \pi_s}{i_l + j_l + 2\pi_s + 2\delta_s},$$
$$E(S_{sl}) = \lambda_{sl} \frac{j_l + \pi_s + \delta_s}{i_l + j_l + 2\pi_s + 2\delta_s},$$
$$E(D_{sl}) = \lambda_{sl} \frac{\delta_s}{i_l + j_l + 2\pi_s + 2\delta_s}.$$

Bayesian models require prior distributions for all unknown parameters. We assumed the total intensities λ_{sl} were drawn from an Exponential distribution with mean 10,000, modeling diffuse prior distributions spread over a wide range. Patient and donor contamination levels were assumed to come from Exponential distributions truncated at an upper bound of 0.2. Pathology studies concluding that contamination levels are probably less than 0.01 and very unlikely to be as high as 0.05 support confidence in 0.2 as a conservative upper bound. The Exponential shape was again meant to be conservative, allowing high probabilities for contamination levels up to 0.2 (in fact this choice hardly differs from a uniform distribution on that interval). The additive standard deviation σ_{add} was taken to have a half-Cauchy distribution with scale parameter 25 (*3*), and the multiplicative variance σ^2_{mult}, a uniform distribution on the interval from 0 to 0.25. Finally, for cancer cells, we need a prior for the number of copies of each allele at each locus, that is, a probability distribution governing the numbers i_l, j_l, and k_l. To check for undesirable sensitivity to this choice we repeated our analysis with the two different distributions *p*1 and *p*2 whose probabilities are shown in Table S2.

		Number of copies										
		0	1	2	3	4	5	6	7	8	9	10
Distribution	*p*1	.10	.25	.18	.13	.10	.08	.06	.04	.03	.02	.01
	*p*2	.07	.80	.07	.03	.01	.006	.003	.001	.0005	.0002	.0001

Table S2. Two prior distributions for allelic copy numbers in cancer cells.

These distributions incorporate the aneuploidy that occurs in cancer cells, so that alleles can be lost or gained. Such losses and gains are less likely in distribution $p2$ than they are in $p1$. In both distributions, increasing copy numbers of 2 through 10 are assigned decreasing probabilities. These $p2$ probabilities decrease more quickly than the $p1$ probabilities, so that $p2$ assigns much smaller probability to high copy numbers than $p1$ does. To avoid duplication we will report results in detail mainly for distribution $p1$; in fact the conclusions turned out to be the same using both distributions, showing stability and lack of sensitivity to these different choices of prior distribution.

Model fitting

The models were fit by Markov chain Monte Carlo (MCMC, e.g., (*4*)), using the software JAGS (*5*). We used two different run lengths for different models: shorter runs of 110,000 iterations with the first 10,000 iterations discarded as "burn-in", and 1,100,000 iterations with the first 100,000 iterations discarded. The shorter runs were used in many initial investigations of new models and also in creating the calibration distributions in the computationally intensive calibration procedure described below. Informal checks of time-series plots of the parameters as well as the deviances (defined as twice the negative log likelihood; smaller deviance indicates better fit) indicated that in fact very little burn-in was needed and the amount discarded was more than enough. The longer runs were used for extra accuracy and to increase certainty that better regions of the parameter space were not being missed. The longer runs were also used in establishing good initial values for the shorter runs in the calibration distributions, and choosing these initial values helped the shorter runs to work well. Fig. S7 shows plots of the deviances obtained by two of the longer runs. They support confidence that the mixing of the Markov chains is satisfactory.

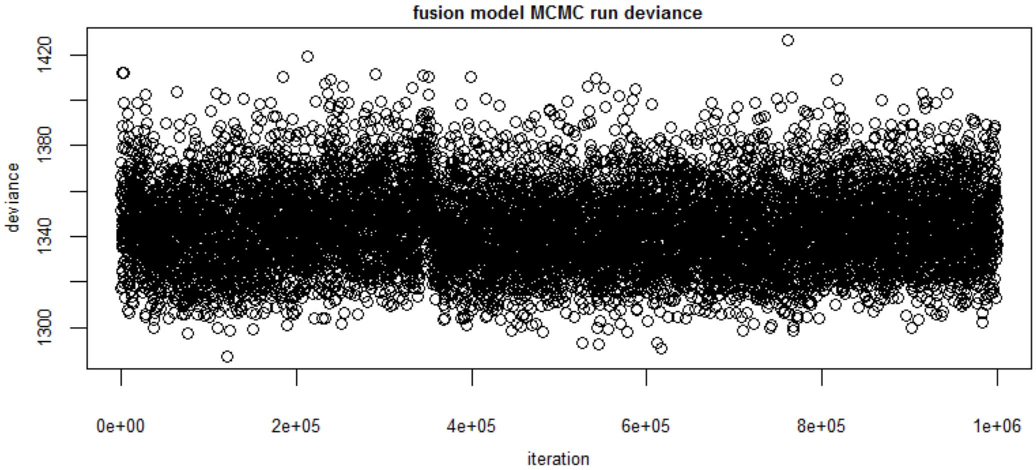

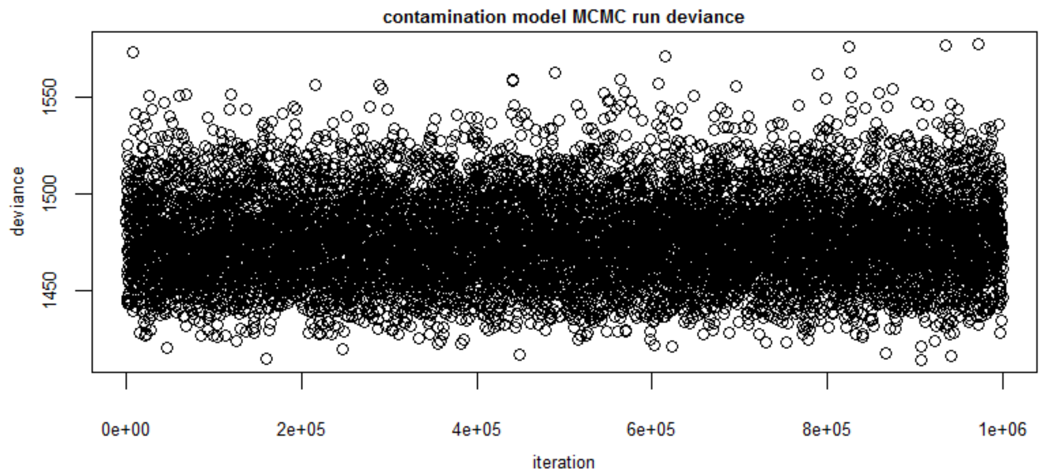

Fig. S7. Plots of deviance (−2 times log likelihood) over MCMC runs of fusion and contamination models.

The result of each MCMC run for a given model is a sample of parameter values approximately from the posterior distribution for that model, and the distributions of these samples are summarized in the following figures. We begin with the fusion model. The next figure shows the posterior samples for the allele counts of the patient-specific, shared, and donor-specific alleles. It suggests, for example, that at the FGA locus, the fusion cells had several copies of the patient-specific and shared alleles, and one copy of the donor specific allele, and at the D13S317 locus, the patient-specific allele was lost in the fusion cell.

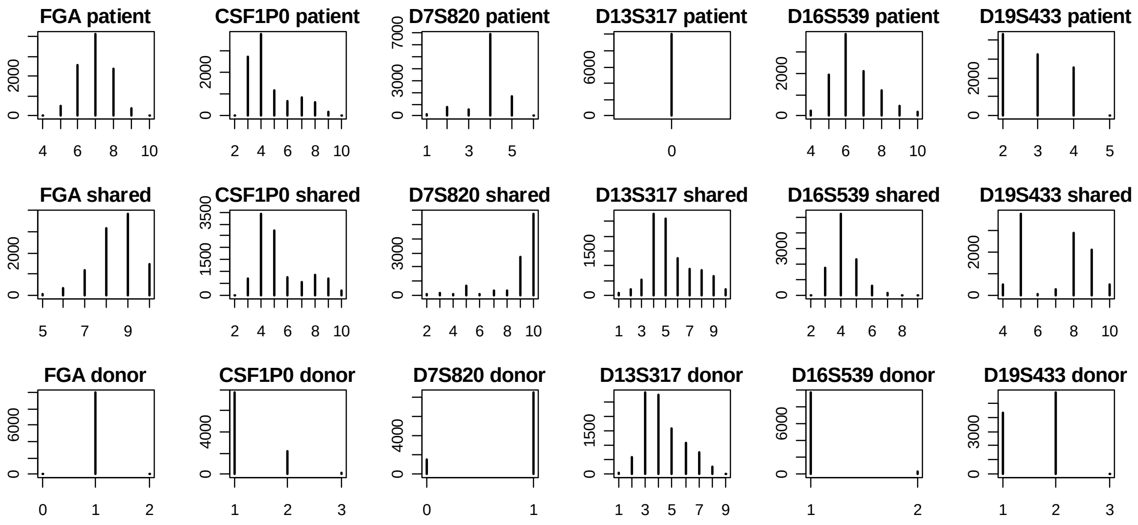

The distributions of the patient contamination levels are summarized in the following histograms.

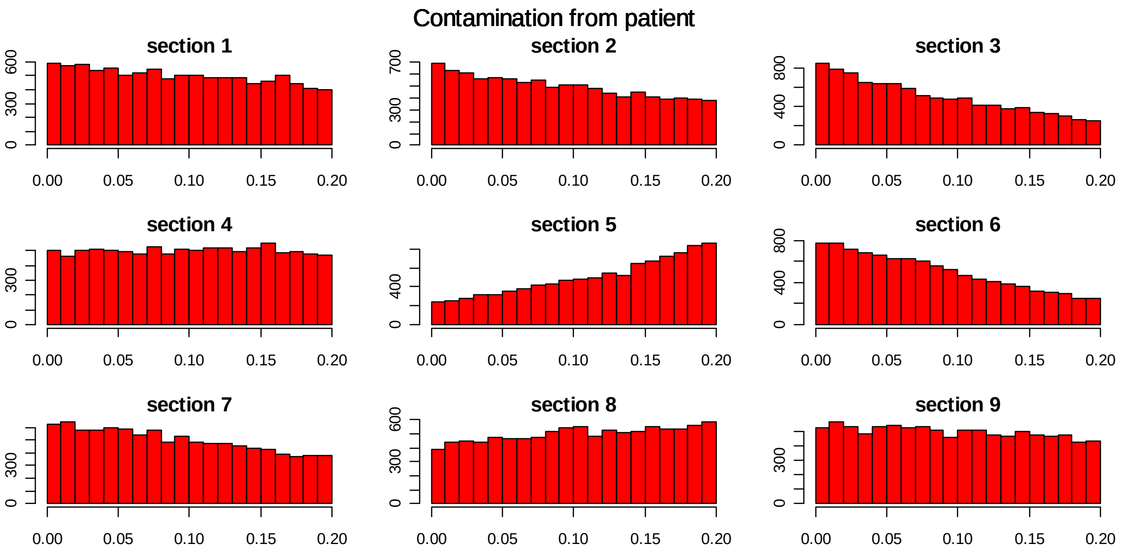

These distributions are not so different from the assumed prior, indicating that the data are not very informative about these parameters. The additive and multiplicative error distributions are described by the following histograms.

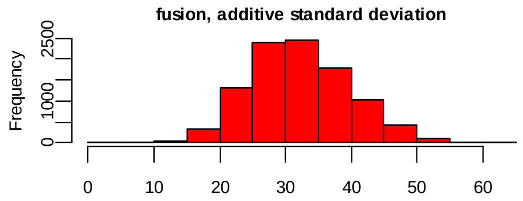

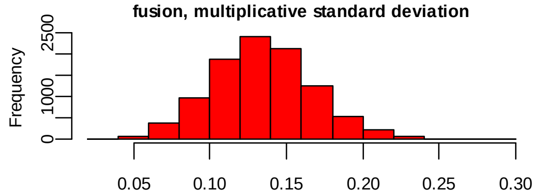

These suggest that the additive standard deviation is around 30 RFU or so, and the multiplicative error standard deviation is on the order of 10 to 15 percent or so.

We calculated standardized Bayesian residuals defined by $\left(Y_{sl} - E(Y_{sl} \mid \theta^{(t)})\right) / \mathrm{SD}(Y_{sl} \mid \theta^{(t)})$, where Y_{sl} is replaced by P_{sl}, S_{sl}, or D_{sl}, and $\theta^{(t)}$ denotes the parameters sampled at iteration t of the MCMC simulation. After doing so, we could check for flagrant violations of the Normality assumptions by plotting these residuals against the quantiles of a standard Normal distribution; Fig. S8 shows this plot for the fusion model. The ideal expectation under perfect Normality in such a plot would be a straight line sloped upwards at 45°, and so Fig. S8 shows that the Normality assumptions appear to be very reasonable.

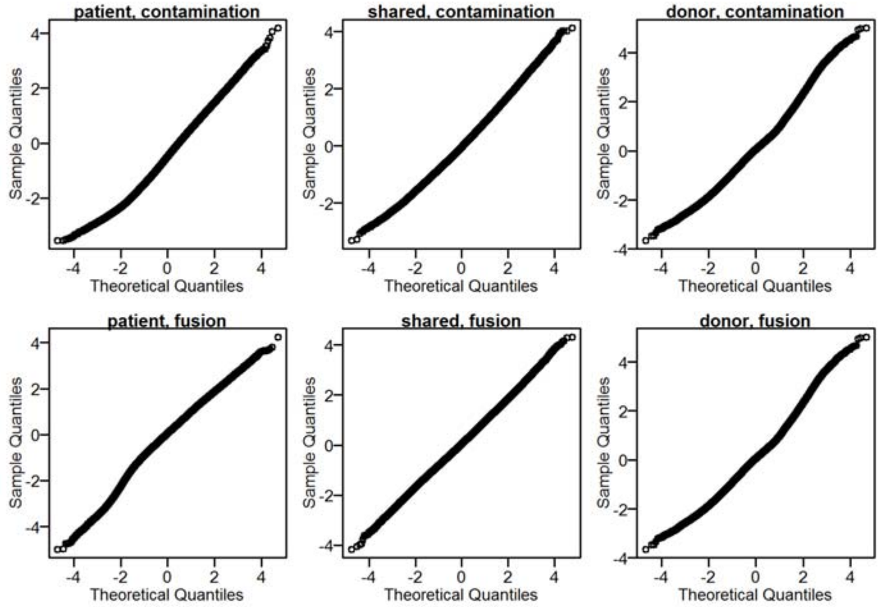

Fig S8. Graphical checks of the Normality distributional assumptions for residuals.

The following plots show corresponding pictures for the contamination model. The allele count distributions and the patient contamination levels are shown in the next two plots.

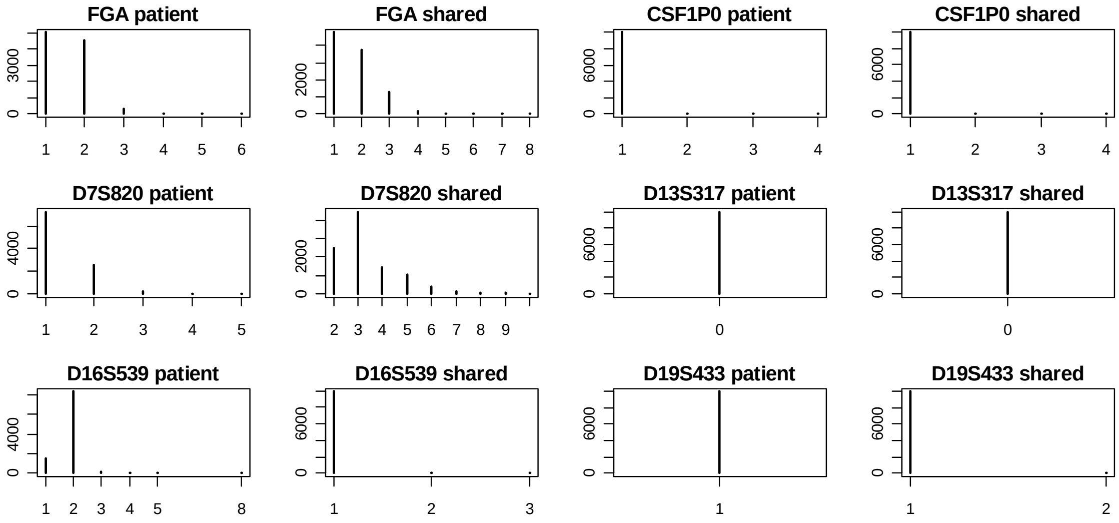

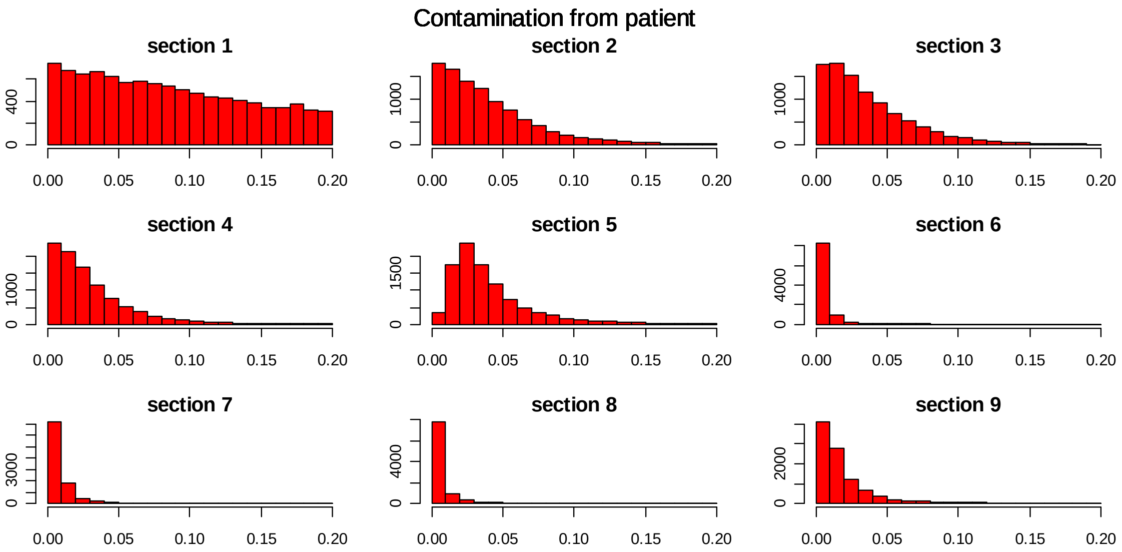

Perhaps most telling are the donor contamination levels δ_s, which came out as follows:

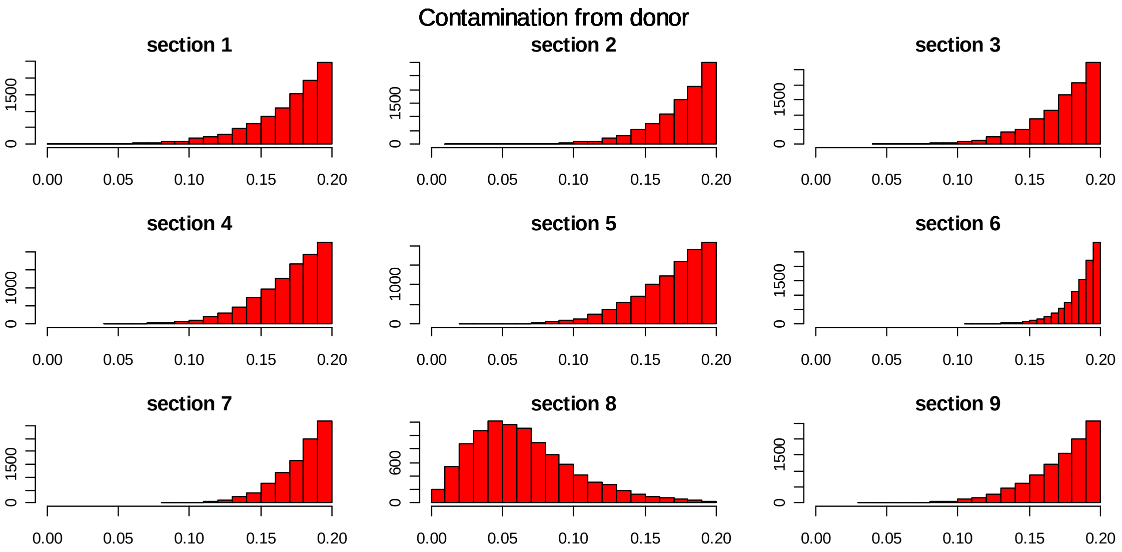

Note that in several of the tumor sections, the fitted model suggests that the donor contamination levels are very high, approaching the assumed upper limit of 0.2. In fact, if the prior distributions had not been constrained by the upper limit of 0.2, the Markov chain would have resorted to even higher to levels of contamination that are much too high to be plausible.

Model comparison

To compare the fits of the contamination and fusion models, a quick first indication was obtained by inspecting the deviances for each model.

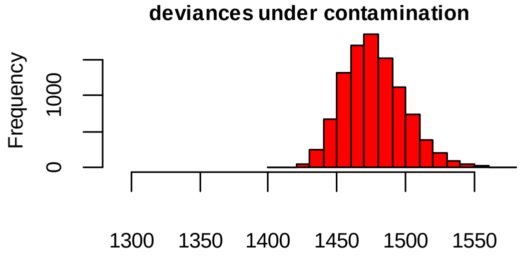

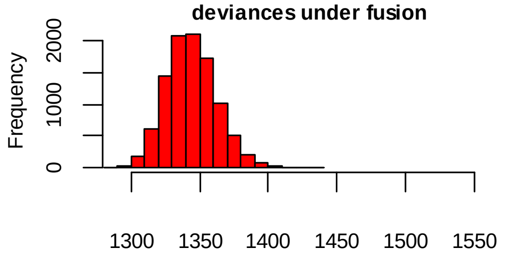

The deviances for the fusion model were clearly smaller than those for the contamination model, indicating a better fit for fusion. In view of the fact that the fusion model has fewer parameters (6 more allele count parameters but 9 fewer contamination parameters) than the contamination model, which is expected to put the fusion model at a disadvantage as far as deviances are concerned, this comparison is all the more striking.

For a probabilistic comparison we turned to the log pseudo-marginal likelihood (LPML, (*6*)), a measure of model fit advocated in the statistical literature (*1*,*2*). We calculated the LPML for both the fusion model and contamination model as fit to our observed data. These two individual values are not particularly meaningful on their own, but the difference (also known as the log pseudo-Bayes factor) is of interest, indicating how much better the fusion model fit our data than the contamination model did. An important advantage of this approach over those based on standard Bayes factors is that the quantities we are computing are much less sensitive to the choice of prior distributions. For our data, the LPML difference turned out to be 71.4, the positive value indicating that the fusion model fit the data better than the contamination model. To assess the statistical significance of the value 71.4, we used a computationally intensive calibration procedure (*7*,*8*), in which 400 random data sets were simulated under the two models, and for each simulated data set, an LPML difference was calculated. The calibration distributions obtained by this procedure are shown in the histograms in Fig. S9, where the red lines show the calculated value 71.4 of this LPML difference for our data.

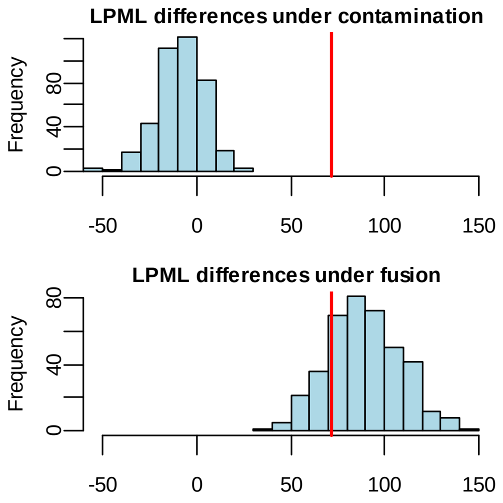

Fig S9. Calibration distributions of LPML differences under contamination and fusion models.

As shown in Fig. S9, under contamination, all 400 of the LPML differences were less than 71.4, leading to an empirical two-sided *P* value bounded by $P < \frac{2}{400} = 0.005$. Thus, LPML differences as high as 71.4 are very rare under the contamination model. In contrast, under the fusion model, a LPML difference of 71.4 was quite typical, being at the 18.5 percentile of the calibration distribution, leading to a *P* value of 0.37. The finding of a small P value for contamination supports the assertion that the contamination model fails to explain our data. In contrast, the much larger P value for fusion indicates that our data is consistent with the fusion model.

Model variations

A number of variations of the above models were considered and evaluated by the same methods. As mentioned above, we tried varying the prior distributions for allele counts in the cancer cells from the distribution $p1$ to the distribution $p2$ defined in Table S2. Compared to $p1$, the distribution $p2$ places more probability on 1 copy and less probability on losses and gains, particularly the higher copy numbers. But the prior $p2$ led to the same conclusions, giving a P value less than 0.005 for contamination and P=0.645 for fusion. Another modification was to replace the Normal error distributions by t distributions, which have longer tails and allow more extreme outliers. We also investigated a simple form of tumor evolution by extending the above models to allow repeat numbers within a single locus to take two different values over the different tumor sections. None of these more complicated models was found to provide a significant improvement over the final models presented above in fitting our data.

REFERENCES

1. J. G. Ibrahim, M. H. Chen, D. Sinha, *Bayesian survival analysis* (Springer Verlag, New York, 2001).

2. R. Christensen, W. Johnson, A. Branscum, *Bayesian ideas and data analysis: An introduction for scientists and statisticians* (CRC Press, Boca Raton, 2010).

3. A. Gelman, Prior distributions for variance parameters in hierarchical models. *Bayesian analysis*. **1**, 515-533 (2006).

4. D. Gamerman, H. F. Lopes, *Markov chain Monte Carlo: stochastic simulation for Bayesian inference* (Chapman & Hall/CRC, 2006) [Vol. 68].

5. M. Plummer, JAGS: A program for analysis of Bayesian graphical models using Gibbs sampling, in *Proceedings of the 3rd International Workshop on Distributed Statistical Computing* Vienna, Austria, 2003.

6. S. Geisser, W. Eddy, A Predictive Approach to Model Selection. *J. Amer. Statistical Assoc*. **74**, 153-160 (1979).

7. P. K. Vlachos, A. E. Gelfand, On the calibration of Bayesian model choice criteria. *Journal of Statistical Planning and Inference* **111**, pp. 223-234 (2003).

8. D. Draper, M. Krnjajić, Bayesian Model Specification: Some problems related to model choice and calibration, in *Applied Methods of Statistical Analysis, Simulations and Statistical Inference* (Novosibirsk, Russia, 2011). pp. 133-142.
